# The case of eculizumab: litigation and purchases by the Brazilian Ministry of Health

**DOI:** 10.11606/s1518-8787.2020054001693

**Published:** 2020-02-12

**Authors:** Rosângela Caetano, Paulo Henrique Almeida Rodrigues, Marilena C Villela Corrêa, Pedro Villardi, Claudia Garcia Serpa Osorio-de-Castro

**Affiliations:** I Universidade do Estado do Rio de Janeiro Instituto de Medicina Social Departamento de Política, Planejamento e Administração em Saúde Rio de JaneiroRJ Brasil Universidade do Estado do Rio de Janeiro. Instituto de Medicina Social. Departamento de Política, Planejamento e Administração em Saúde. Rio de Janeiro,RJ, Brasil; II Universidade do Estado do Rio de Janeiro Instituto de Medicina Social Departamento de Políticas e Instituições de Saúde Rio de JaneiroRJ Brasil Universidade do Estado do Rio de Janeiro. Instituto de Medicina Social. Departamento de Políticas e Instituições de Saúde. Rio de Janeiro, RJ, Brasil; III Fundação Oswaldo Cruz Escola Nacional de Saúde Pública Sergio Arouca Departamento de Política de Medicamentos e Assistência Farmacêutica Rio de JaneiroRJ Brasil Fundação Oswaldo Cruz, Escola Nacional de Saúde Pública Sergio Arouca. Departamento de Política de Medicamentos e Assistência Farmacêutica. Rio de Janeiro, RJ, Brasil

**Keywords:** Hemoglobinuria, Paroxysmal, drug therapy, Drug Costs, Orphan Drug Production, legislation & jurisprudence, Health’s Judicialization, Public Expenditures on Health, National Drug Policy

## Abstract

**OBJECTIVES:**

This study examined the purchases of eculizumab, a high-cost monoclonal antibody used in the treatment of rare diseases by Brazilian federal agencies, in terms of purchased quantities, expenditures, and prices.

**METHODS:**

Eculizumab purchases made between March 2007 and December 2018 were analyzed, using secondary data extracted from the Federal Government Purchasing System (SIASG in Portuguese). The following aspects were assessed: number of purchases, purchased quantities, number of daily doses defined per 1,000 inhabitants per year, annual expenditures, and prices. The prices were adjusted by the National Broad Consumer Price Index for December 2018. Linear regression was used for trend analysis.

**RESULTS:**

All acquisitions by federal agencies were made by the Brazilian Ministry of Health. The purchases began in 2009 with tender waiver to comply with legal demand. There was an increasing trend in the number of purchases and quantities acquired over time. Two hundred and eighty-three purchases were made, totaling 116,792 units purchased, 28.2% of them in 2018. The adjusted total expenses summed more than R$ 2.44 billion. After market approval by the Brazilian Health Regulatory Agency, the weighted average price fell approximately 35%, to values under the Medicines Market Chamber of Regulation established prices.

**CONCLUSION:**

Eculizumab represented extremely significant expenditures for the Brazilian Ministry of Health during the period. All purchases were made to meet demands from lawsuits, outside the competitive environment. The market approval of eculizumab promoted an important price reduction. This study indicates the relevance of licensing and the need for permanent monitoring and auditing of drug purchases to meet legal demands.

## INTRODUCTION

Eculizumab is a high-cost drug indicated to ease adult and pediatric patients’ complications with paroxysmal nocturnal hemoglobinuria (PNH) and atypical hemolytic uremic syndrome (aHUS). The PHN is an acquired disorder of hematopoietic stem cells, which is characterized by a highly variable clinical course, including hemolytic anemia, bone marrow failure, and thromboembolic phenomena, with complement-mediated manifestations^1^ . The aHUS is a clinical entity marked by the presence of thrombocytopenia, microangiopathic hemolytic anemia, and progressive kidney damage, resulting from mutations in genes that control the complement system^2^ .

Both are considered rare diseases because they affect up to 65 people in 100,000 individuals^3^ . Clinically significant PNH affects one to ten individuals per one million, although this number may be underestimated as some patients may remain undiagnosed because of the heterogeneous spectrum of clinical manifestations and diagnostic difficulties^4 , 5^ . The aHUS frequency is also low, even though it is difficult to determine its exact incidence^6^ . In Brazil, the prevalence of the two conditions is unknown^7^ .

Eculizumab is a humanized monoclonal antibody that blocks the activation of the terminal complement C5 and prevents the formation of Complement Component 5a (C5a) and C5b-9, reducing the need for blood transfusions and the thrombolysis risk^8^ . The drug was registered to PNH in the U.S. Food and Drug Administration (FDA) and the European Medicines Agency (EMA) in 2007, with a record interval of just three months. In both cases, the approval happened because of prerogatives regarding the so-called “orphan drugs” based on small clinical trials^9 , 10^ . The PNH registration in Canadian and Japanese health agencies was obtained, respectively, in 2009 and 2010.

In Brazil, market approval was only asked by the manufacturer (Alexion Pharmaceuticals Inc.) in March 2015, being granted by the Brazilian Health Regulatory Agency (ANVISA) on March 13, 2017^11^ . The price was established by the Brazilian Chamber of Drug Market Regulation (CMED) in October 2017, with maximum retail selling price to the government (PMVG), which corresponds to the price ceiling for any drug purchased by judicial decision, set at R$ 13,614.80 (with 0% Brazilian state excise tax) per 300 mg vial^a^. The treatment of an adult with PNH at the dose defined in the leaflet would correspond to the use of 74 vials in the first treatment year and then 72 vials/year in the second year and onwards, for life. According to data from the Brazilian Federal Attorney General’s Office (AGU), spending on health litigation within the Union totaled more than R$ 1.325 billion in 2016, representing an increase of more than 5,000% compared to the R$ 26 million spent in 2007. Eculizumab has been litigated in Brazil for several years and this modality has provided 100% of the public provision. In 2016 alone, eculizumab legal purchases totaled R$ 624,621,563.43, representing the main item of expenditures and 41.2% of the resources involved in drug litigation^12^.

In 2018, the Secretariat of Science, Technology and Strategic Inputs (SCTIE) of the Brazilian Ministry of Health (MS) submitted a request for the incorporation of eculizumab to the National Committee for Health Technology Incorporation of the Brazilian Unified Health System (Conitec). The drug was assessed and incorporated only for the PNH treatment, from the publication no. 77, of December 14, 2018^13^ .

The drug’s high costs, its significant participation in expenses with health litigation, its economic impact on the Brazilian Ministry of Health expenditures, and the factors involved in its market approval, price, and incorporation by the Brazilian Unified Health System (SUS) highlight the importance of studying the eculizumab purchases made by federal agencies from 2007 to 2018, in terms of quantities purchased, expenditures, and prices.

## METHODS

This is an exploratory study with a quantitative approach, focusing on the purchases of eculizumab performed by agencies of the federal public administration from January 2007 to December 2018. The choice of time interval intended to consider the year of the initial market approval by the health agencies of the United States and Europe, until 2018, when the drug was already licensed in Brazil and had its prices established by CMED.

The following data were extracted from the records of the Federal Government Purchasing System (SIASG), controlled by the Brazilian Ministry of Planning, Budget and Management (MP): number of purchases, quantities purchased, type of acquisition, federal agency responsible for the purchases, contracted supplier, unit prices, amounts of contracted expenditures, and justifications for purchases during each year of the period.

To enable comparability between the years, the unit acquisition prices extracted from SIASG were adjusted for December 2018, applying the annual variation of the National Consumer Price Index (IPCA), according to Law no. 10,742 of 2003, which defined this index for adjustment in drug prices in Brazil^14^ .

In order to assess the historical behavior of drug purchases, trend analyses were performed using linear regression.

Eculizumab purchases can be understood as a proxy for utilization, in this case, consumption. For this analysis, two estimates were made. The number of people potentially treated per year was estimated by dividing the number of 300 mg vials purchased by the number of required vials to treat an adult patient per year (72 vials). The defined daily dose (DDD) was also used, an international measure of consumption recommended by the World Health Organization, which is not strictly related to the actual dose and represents the average dose for a 70 kg adult patient^15^ . The DDD standardization allows comparability. For population estimates, this indicator is parameterized as the number of DDD/1,000 inhabitants/day; in this case, it was considered appropriate to present the number of DDD/1,000 inhabitants/year^16^ . Data from the Brazilian population in the study years were obtained from the Brazilian Institute of Geography and Statistics (IBGE)^17^ .

All data obtained from SIASG were compiled to a database specifically built for this purpose, and statistical calculations, including trend analyses, were performed using the Microsoft Excel softwareⓇ.

Access to SIASG data is public, in accordance with the Brazilian Access to Information Act; thus, not requiring the prior submission of the study to a research ethics committee.

## RESULTS

The mapping of the drug purchases within SIASG showed that the first eculizumab acquisitions occurred in February 2009, two years after the first international market approval license was issued. In the period, all federal eculizumab purchases were performed by the Department of Logistics in Health of the Brazilian Ministry of Health, all exempt from bidding and always with the justification of compliance with legal demands.

The number of purchases and quantities acquired over time showed an increasing trend. Twenty-eight purchases were made in the period, totaling 116,792 vials, 28.2% of which were purchased in 2018 ( Table 1 ). It is also observed that 2017 experienced a significant drop in the number of purchases and in number of purchased dosage forms (13,721).

**Table 1:** Number of federal purchases and of dosage forms of eculizumab (300mg vial), 2009–2018.

Year	Number of purchases	Number of dosage forms acquired	

		Quantity	% Total
2009	2	145	0.12
2010	4	180	0.15
2011	12	754	0.65
2012	16	2,166	1.85
2013	69	8,296	7.10
2014	50	14,061	12.04
2015	57	19,206	16.44
2016	54	25,386	21.74
2017	4	13,721	11.75
2018	15	32,877	28.15
**Total**	**283**	**116,792**	**100.00**

Note: There were no federal purchases in 2017 and 2018.

**Figure 1:**
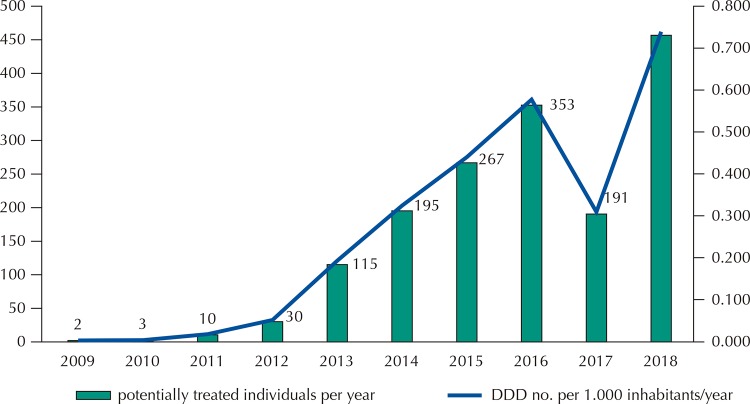
Estimates of number of treated patients per year and number of DDDs per 1.000 inhabitants/year in federal eculizumab purchases. 2009–2018. DDD: defined daily dose

Figure 1 shows the relationship between the number of dosage forms acquired for potentially treated patients and the number of DDD/1,000 inhabitants/year, verifying proportionality and progressive trend increase of both over time.

**Figure 2:**
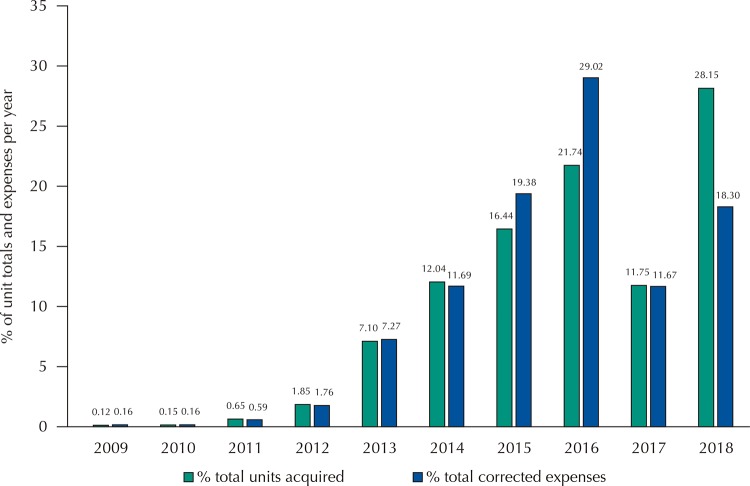
Proportions of units acquired and total expenses adjusted with the eculizumab drug in federal purchases during the period 2009–2018. second year of purchase.

The expenses between 2009 and 2018 totaled R$ 2.1 billion in current values and R$ 2.44 billion in adjusted value by the December 2018 IPCA ( Table 2 ). Despite accounting for more than a quarter of all dosage forms acquired in the period, 2018 corresponded to only 18.3% of the expenditures in the interval studied ( Figure 2 ).

**Table 2:** Expenses contracted in current and adjusted values regarding federal eculizumab purchases (300 mg vial), 2009–2018.

Year	Contracted expenses current amounts (R$)	Adjusted contract expenses (R$)	% adjusted total expenses
2009	2,219,023.25	3,924,941.63	0.16
2010	2,366,823.72	4,000,091.69	0.16
2011	8,979,795.84	14,330,935.34	0.59
2012	28,783,166.97	43,131,746.38	1.76
2013	125,450,088.18	177,631,768.91	7.27
2014	213,729,757.88	285,742,220.30	11.69
2015	376,945,601.92	473,604,100.41	19.38
2016	624,621,376.84	709,126,486.07	29.02
2017	267,111,457.23	285,303,416.91	11.67
2018	447,156,839.74	447,156,839.74	18.30
**Total**	**2,097,363,931.57**	**2,443,952,547.39**	**100.00**

^a^ Prices were adjusted by the National Broad Consumer Price Index for December 2018

**Figure 3:**
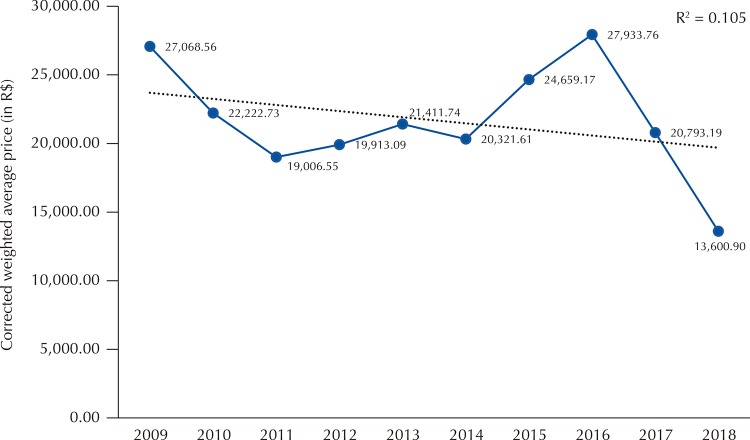
Adjusted weighted average price (in R$) practiced in federal eculizumab purchases (300 mg vial). 2009-2018. Note: Unit prices were adjusted by the National Broad Consumer Price Index for December 2018.

Until registration in 2017, there was a great variation in the weighted average price (WAP) paid per dosage form, which reached R$ 27,933.76 in 2016 ( Figure 3 ). The two purchases made before obtaining market approval at ANVISA in 2017 (10,733 units, 78% of the total purchased in the year) presented a R$ 21,055.61 WAP in current values. The two acquisitions made after market approval and after the setting of the maximum selling price to the government (totaling 2,988 vials) presented a lower WAP (R$ 13,515.01) than the one established by the CMED in October 2017 (R$ 13,614.90), showing an important impact on price reduction (-35.8%). All purchases in 2018 presented a lower WAP (R$ 13,600.90) than the amount per vial defined by CMED in April 2018 (R$ 13,889.35).

## DISCUSSION

Eculizumab has been frequently mentioned in the pages of newspapers and media venues in recent years. The drug has already been described as the most expensive in the world, with an approximate cost of $410,000 per patient/year in the U.S. in 2010^18^ . In Brazil, the cost of treating a single patient per year, who obtained treatment via legal means, exceeded R$ 800,000 in 2012^19^ .

Since 2009, eculizumab has been bought in the country in great quantities and its expenditures show a growing trend over time. However, 2017 presented an important drop in the number of purchases (from more than 50 purchases in the previous three years to only four in that year) and in purchased dosage forms (a -49.5% reduction when compared to the quantity acquired in 2016). The data available in SIASG do not allow the study to expand the understanding of this abrupt decline, but two facts may have contributed. In 2017, the Brazilian Federal Police launched the *Operação Cálice de Hígia* (Operation Bowl of Hygieia), with the aim of investigating possible fraud regarding the litigation of drugs for rare diseases, including SolirisⓇ,eculizumab’s trade name^b^. That same year, the MS undertook the auditing of the drug purchasing process. Of the 414 people who had court decisions to receive the drug in 2017, 28 were not located; five did not reside at the informed address; six refused to provide information; and 13 had already passed away. Graver still: about half of the patients did not present evidence of diagnosis of the disease and, still had been receiving eculizumab by court decision^c^.

On the other hand, in terms of the number of purchases and quantities, growth resumed in 2018, and the year accounted for 28.2% of the total number of dosage forms acquired over the period. Part of this increase may be a backlash to the already mentioned drop in 2017, since it is a chronic drug that is used for life. It is also not uncommon after the broad disclosure of the drug’s market approval to induce an increase in prescribing, in this case still under the aegis of litigation, since the drug had not been included in the SUS funding list.

This inclusion only occurred at the end of December 2018 and, until the date of manuscript preparation, the drug awaited the development of a clinical protocol by the MS to be regularly provided to patients. Over the years studied and even without the presence of an active market license until March 2017, the access to the drug has always occurred via legal demands, in individual actions that have been responsible for extremely significant expenditures.

The literature shows many examples of how litigation has been used as a strategy to access unlicensed drugs in Brazil^20^ . Evidence suggests that pharmaceutical companies may use relationships with patient advocacy groups and health professionals to expand market share by litigation, eventually forcing the incorporation of the drug into the health system^23 , 24^ . A study of 514 lawsuits that demanded the drug and had the MS as a defendant, between 2010 and 2016, showed that 376 (73%) originated in the Federal District and 46 (9%) in the state of São Paulo. Only a single law firm was responsible for 361 lawsuits (70%). The proportions of prescriptions originating from private physicians and SUS are similar (respectively, 32.4% and 31.2%), drawing attention to the fact that in 27.1% of the lawsuits there was no record of the prescriber’s name^25^ .

Eculizumab is considered an “orphan drug”, which gives it a set of specificities in terms of sanitary licensing, prices, and access for payment or reimbursement by health systems. The designation of “orphan” is associated with drugs developed for the treatment of diseases that would presumably provide little economic return on investment in research and development (R&D) made by the pharmaceutical industry or patent holders. Low profitability may be due to the low prevalence or incidence of the disease in a population, which would make the drug market small and unprofitable, or to therapeutic focus for prevalent conditions in less developed countries devoid of resources for payment of the price that would represent profit for the industry. As a result, many legislations were successively created in the United States (1983), Japan (1993), Australia (1998), and Europe (2000) to encourage the development of orphan drugs^26^ .

The high price paid for eculizumab by the MS and its variation over time draws attention. Use directed to a rare disease is often reported as a reason for the high price of the drug, with the justification that it incurs R&D costs similar to those aimed at common diseases, but with fewer potential users to ensure the return on investments. However, this justification has been questioned. The sale of drugs for rare diseases has sometimes become more profitable than traditional drugs because of the granting of various government benefits, such as market exclusivity for longer periods, tax incentives, economic support for the development of specific research, and market approval in a shorter timeframe and with less demanding scientific evidence criteria^26^ .

However, the cost of clinical trials for “orphan drugs” is much lower than for drugs for other diseases, since the number of patients involved is much smaller. Small clinical studies and absence of alternative treatments put these “orphan drugs” to the advantage when submitted to regulatory reviews. Few patients and lack of competition also determine the need for lower investments in marketing. Moreover, given their high cost, these drugs are generally funded by governments or insurers^29^ . As a result, pharmaceutical companies with authorization to market orphan products can be more profitable than those without this type of product in their portfolios. Between 2000 and 2012, companies that sold “orphan drugs” had a return on investment 9.6% higher than manufacturers of “non-orphaned” drugs^30^ .

The request for market approval of a drug in Brazil and, subsequently, the request for pricing by CMED are exclusively manufacturer attributions. Until 2017, the absence of market approval and price determined that the Brazilian Ministry of Health was obliged to import it at any price charged by the supplier, burdened by the entire transport and distribution costs to meet the lawsuit demand for eculizumab.

Although the drug was the subject of purchases to meet litigation demands for a long time, the company did not request market approval in the country for almost nine years after its introduction in American and European markets, even considering the size of the Brazilian population and the prioritization of analysis given by ANVISA to drugs for rare diseases^31^ . The drug was only registered in 2015, when the patent protection of eculizumab coincidentally expired in the country.

Without market approval there was no established price for sale to the government and it could not be submitted to assessment by Conitec. But it continued to be purchased to treat few patients via litigation, always at high prices, and with bidding waiver. In other words, until 2015 the company’s profit was assured since the producer fully defined and controlled the sales prices.

These aspects clarify the effect of market approval on the verified price reduction, greater than 35% in 2017, and the maintenance of this decrease also in 2018, even if at a smaller level. This was not a consequence of incorporation by Conitec which only took place in December 2018; incorporation supposedly schedules needs and acquisitions, allowing for regular and centralized purchases, economies of scale, and the use of state purchasing power for negotiations and lower prices^32^ . Major drops may still occur since one of the explicit conditions in the decision to incorporate the drug into SUS is the “negotiation for significant price reduction”^7^ .

Another factor with future reducing impact potential is the possibility of establishing a productive development partnership (PDP) for technological transfer and local production, since the drug is no longer under patent. On this regard, it is noteworthy that eculizumab was part of the annual list of strategic products for SUS, with a view to the formation of a PDP published in the MS Ordinance, no. 704, March 2017^33^ . However, the drug does not appear in the MS/GM Ordinance no. 731, March 26, 2018,^34^ which presents the list of approved projects, or in the list of approved PDP proposals made available on the DECEIIS/SCTIE^d^ page, leading to the assumption that no proposals were submitted by interested companies.

Another factor with future reducing impact potential is the possibility of establishing a productive development partnership (PDP) for technological transfer and local production, since the drug is no longer under patent. On this regard, it is noteworthy that eculizumab was part of the annual list of strategic products for SUS, with a view to the formation of a PDP published in the MS Ordinance, no. 704, March 2017^33^ . However, the drug does not appear in the MS/GM Ordinance no. 731, March 26, 2018,^34^ which presents the list of approved projects, or in the list of approved PDP proposals made available on the DECEIIS/SCTIE^d^ page, leading to the assumption that no proposals were submitted by interested companies.

Although a drop in lawsuits is expected after the incorporation into the SUS, this will not necessarily occur. Incorporation establishes an official market, however, with lower prices than those practiced in litigation and only for the defined indication (PNH). It is still possible to appeal to the judicial route for cases that do not meet the criteria shown in the clinical protocol, as well as for other registered (aHUS) and unregistered indications in the country,^35 , 36^ keeping this profit filter alive for the company. Monitoring of purchases and of prices may be useful to better clarify future behavior regarding this drug.

The estimated utilization values were independently estimated (inferred by the number of potential treatments or estimated by DDD/1,000 inhabitants/year) and are consistent, with a progressive trend of growth over time. There are no international DDD utilization data but estimates will allow comparability over time^37^ . The requirements for patient care and treatment only in Brazilian National Clinical Research Network hospitals and the mandatory recording of clinical and pharmaceutical data in the SUS may bring forth more reliable utilization information^7^ .

This study has some limitations. Although SIASG shows contracted purchase data and not finalized purchases, it is reasonable to assume that, in the case of drugs purchased for legal demands, these purchases would have been made effective. The aggregated data shown in SIASG also do not show the number of treated patients or of new patients treated each year, the indications of treatment underlying purchases from litigation or the expansion of post-incorporation coverage. Finally, the research interval fails to identify the impact of incorporation, which occurred at the end of the studied period in respect to quantities and prices.

The growth of purchased volumes indicates expansion of eculizumab use over time. The high associated expenditures have a significant financial impact on the provision of drugs by the Brazilian Ministry of Health, especially because until the end of 2018 100% of access to the drug was linked to litigation. Furthermore, the price variation before market approval also reinforces the importance of the licensing requirement for manufacturers who wish to sell drugs in the domestic market. Finally, the study shows the need for permanent monitoring and auditing of drug purchases to meet lawsuits, especially those for high-cost drugs.

## Footnotes

a
Ministério da Saúde (BR), Agência Nacional de Vigilância Sanitária, Câmara de Regulação do Mercado de Medicamentos-CMED. Preços máximos de medicamentos por princípio ativo, para compras públicas Preço Fábrica (PF) e Preço Máximo de Venda ao Governo (PMVG): tabela atualizada em 20/10/2017 [citado 27 mar 2019]. Disponível em: http://portal.anvisa.gov.br/documents/374947/2829072/LISTA_CONFORMIDADE_GOV_2017-10-20.pdf/d25373b5-3224-4ef5-b62f-9367de5fb8d0

b
Reuters. PF investiga fraude bilionária ligada a remédio para doença rara. Exame, 8 de maio de 2017. [citado 28 mar 2019]. Disponível em: https://exame.abril.com.br/brasil/pf-investiga-fraude-bilionaria-ligada-a-remedio-para-doenca-rara/

c
Ministério da Saúde (BR), Secretaria de Ciência, Tecnologia e Insumos Estratégicos, Departamento do Complexo Industrial e Inovação em Saúde. Propostas de Projeto de Parcerias para o Desenvolvimento Produtivo não aprovadas. Brasília, DF; 2017 [citado 4 abr 2019]. Disponível em: http://portalarquivos2.saude.gov.br/images/pdf/2018/junho/18/Propostas-de-projeto-de-PDP-nao-aprovadas-2017.pdf

d
Ministério da Saúde (BR), Secretaria de Ciência, Tecnologia e Insumos Estratégicos, Departamento do Complexo Industrial e Inovação em Saúde. Propostas de Projeto de Parcerias para o Desenvolvimento Produtivo não aprovadas. Brasília, DF; 2017 [citado 4 abr 2019]. Disponível em: http://portalarquivos2.saude.gov.br/images/pdf/2018/junho/18/Propostas-de-projeto-de-PDP-nao-aprovadas-2017.pdf
