# Supplementary material for: The case of eculizumab: litigation and purchases by the Brazilian Ministry of Health
Source: Rev Saude Publica. 2020 Feb 12;54:22. doi: 10.11606/s1518-8787.2020054001693 (PMC7017980; doi:10.11606/s1518-8787.2020054001693)
Supplement: Translation

# Translation: O caso do eculizumabe: judicialização e compras pelo Ministério da Saúde

**Authors:** Rosângela Caetano, Paulo Henrique Almeida Rodrigues, Marilena C Villela Corrêa, Pedro Villardi, Claudia Garcia Serpa Osorio-de-Castro

**Affiliations:** I Brasil Universidade do Estado do Rio de Janeiro. Instituto de Medicina Social. Departamento de Política, Planejamento e Administração em Saúde. Rio de Janeiro,RJ, Brasil; II Brasil Universidade do Estado do Rio de Janeiro. Instituto de Medicina Social. Departamento de Políticas e Instituições de Saúde. Rio de Janeiro, RJ, Brasil; III Brasil Fundação Oswaldo Cruz, Escola Nacional de Saúde Pública Sergio Arouca. Departamento de Política de Medicamentos e Assistência Farmacêutica. Rio de Janeiro, RJ, Brasil

**Keywords:** Hemoglobinúria Paroxística, tratamento farmacológico, Custos de Medicamentos, Produção de Droga sem Interesse Comercial, legislação & jurisprudência, Judicialização da Saúde, Gastos Públicos com Saúde, Política Nacional de Medicamentos

## Abstract

**OBJETIVOS:**

O estudo examinou as aquisições de eculizumabe, um anticorpo monoclonal de alto custo utilizado no tratamento de doenças raras, pelos órgãos federais brasileiros, em termos das quantidades compradas, gastos e preços.

**MÉTODOS:**

Foram analisadas compras de eculizumabe realizadas entre março de 2007 e dezembro de 2018, por meio de dados secundários extraídos do sistema de compras do governo federal (Siasg). Foram examinados o número de compras, quantidades adquiridas, número de doses diárias definidas por 1.000 habitantes por ano, gastos anuais e preços praticados. Os preços foram corrigidos pelo índice nacional de preços ao consumidor amplo para dezembro de 2018. Regressão linear foi utilizada para análises de tendência.

**RESULTADOS:**

Todas as aquisições por órgãos federais foram realizadas pelo Ministério da Saúde. As compras se iniciaram em 2009, sendo efetuadas por dispensa de licitação e para atendimento de demanda judicial. Houve tendência crescente no número de compras e quantidades adquiridas ao longo do tempo. Foram realizadas 283 compras, totalizando 116.792 unidades adquiridas, 28,2% compradas em 2018. Os gastos totais contratados corrigidos somaram mais de R$ 2,44 bilhões. Após a aprovação do registro pela Agência Nacional de Vigilância Sanitária, o preço médio ponderado caiu aproximadamente 35%, para valores abaixo dos preços estabelecidos pela Câmara de Regulação do Mercado de Medicamentos.

**CONCLUSÃO:**

O eculizumabe representou gastos extremamente significativos para o Ministério da Saúde no período. Todas as compras foram feitas para atendimento de demandas judiciais, fora do ambiente competitivo. Seu registro promoveu queda importante nos preços praticados. O estudo aponta a relevância do registro sanitário e da necessidade de monitoramento e auditoria permanentes das compras de medicamentos para atendimento de demandas judiciais.

## INTRODUÇÃO

O eculizumabe é um medicamento de alto custo, indicado para amenizar complicações de pacientes adultos e pediátricos com hemoglobinúria paroxística noturna (HPN) e síndrome urêmico-hemolítica atípica (SHUa). A HPN é um distúrbio adquirido das células-tronco hematopoiéticas, que se caracteriza por curso clínico altamente variável, incluindo anemia hemolítica, falência da medula óssea e fenômenos tromboembólicos, com as manifestações mediadas por complemento^1^ . Já a SHUa é uma entidade clínica demarcada pela presença de trombocitopenia, anemia hemolítica microangiopática e dano renal progressivo, decorrente de mutações nos genes que controlam o sistema complemento^2^ .

Ambas são consideradas doenças raras, porque afetam até 65 pessoas em cada 100 mil indivíduos^3^ . A HPN clinicamente significativa afeta de um a dez indivíduos por milhão, embora esse número possa estar subestimado, porque alguns pacientes podem permanecer não diagnosticados devido ao espectro heterogêneo de manifestações clínicas e a dificuldades diagnósticas^4 , 5^ . A frequência da SHUa é igualmente baixa, ainda que a incidência exata seja difícil de determinar^6^ . Números sobre a prevalência das duas condições no Brasil são desconhecidos^7^ .

O eculizumabe é um anticorpo monoclonal humanizado que bloqueia a ativação do complemento terminal C5 e previne a formação de C5a e C5b-9, reduzindo a necessidade de transfusões sanguíneas e risco de trombólise^8^ . O fármaco foi registrado para a HPN na *Food and Drug Administration* (FDA) americana e na *European Medicines Agency* (EMA) em 2007, com intervalo de registro de apenas três meses. Em ambas, a aprovação se deu utilizando as prerrogativas relacionadas aos chamados “medicamentos órfãos”, com base em pequenos ensaios clínicos^9 , 10^ . O registro da HPN nas agências sanitárias canadense e japonesa foi obtido, respectivamente, em 2009 e 2010.

No Brasil, o registro do medicamento só foi solicitado pelo fabricante (Alexion Pharmaceuticals Inc.) em março de 2015, sendo concedido pela Anvisa em 13 de março de 2017^11^ . Teve preço estabelecido pela Câmara de Regulação do Mercado de Medicamentos (CMED) em outubro de 2017, com preço máximo de venda ao governo (PMVG), que corresponde ao teto de preço para compra de qualquer medicamento adquirido por força de decisão judicial, estabelecido em R$ 13.614,80 (com ICMS de 0%) por frasco-ampola de 300 mg^a^. O tratamento de um adulto com HPN, na dose definida em bula, corresponderia à utilização de 74 frascos-ampola no primeiro ano e 72 frascos/ano do segundo ano em diante, por toda a vida.

Segundo dados da Advocacia Geral da União (AGU), os gastos com a judicialização da saúde no âmbito da União somaram mais de R$ 1,325 bilhões em 2016, representando aumento de mais de 5.000% em relação aos R$ 26 milhões dispendidos em 2007. O eculizumabe vem sendo objeto de judicialização no Brasil há vários anos, e essa modalidade tem fornecido 100% da provisão pública. Apenas em 2016, compras judiciais do eculizumabe totalizaram R$ 624.621.563,43, representando o principal item de gastos e 41,2% dos recursos envolvidos com a judicialização de medicamentos^12^ .

Em 2018, a Secretaria de Ciência, Tecnologia e Insumos Estratégicos (SCTIE) do Ministério da Saúde (MS) submeteu uma solicitação de incorporação do eculizumabe à Comissão Nacional de Incorporação de Tecnologias do SUS (Conitec). O medicamento foi avaliado e incorporado apenas para tratamento da HPN, a partir da publicação da Portaria nº 77, de 14 de dezembro de 2018^13^ .

Os altos custos do medicamento, sua participação expressiva nos dispêndios com a judicialização da saúde, seu impacto econômico nos gastos do MS e os fatores envolvidos no registro, preço e sua incorporação ao Sistema Único de Saúde (SUS) tornam relevante estudar as compras do eculizumabe realizadas por órgãos federais de 2007 a 2018, em termos de quantidades adquiridas, gastos e preços praticados.

## MÉTODOS

Foi empreendido um estudo exploratório, de abordagem quantitativa, com foco nas aquisições do eculizumabe realizadas pelos órgãos da administração pública federal durante o período de janeiro de 2007 até dezembro de 2018. A escolha do intervalo temporal pretendeu contemplar desde o ano do registro inicial nas agências sanitárias dos Estados Unidos e Europa até 2018, quando o medicamento já se encontrava registrado no Brasil e com preços estabelecidos pela CMED.

O número de compras, quantidades adquiridas, tipo de aquisição, órgão federal responsável pelas compras, fornecedor contratado, preços unitários, valores dos gastos contratados e as justificativas de compra de cada ano do período foram extraídos dos registros do Sistema Integrado de Administração de Serviços Gerais (Siasg), de responsabilidade do Ministério do Planejamento, Orçamento e Gestão.

Com vistas a possibilitar a comparabilidade entre os anos, os preços unitários de aquisição extraídos do Siasg foram corrigidos para dezembro de 2018, empregando-se a variação anual do índice nacional de preços ao consumidor amplo (IPCA), em concordância com a Lei nº 10.742 de 2003, que definiu esse índice para fins de ajuste nos preços dos medicamentos no Brasil^14^ .

Com a finalidade de avaliar o comportamento histórico das compras do medicamento, foram elaboradas análises de tendência, utilizando regressão linear.

As compras do eculizumabe podem ser compreendidas como *proxy* de utilização, no caso, consumo. Para esta análise, foram realizadas duas estimativas. O quantitativo de pessoas potencialmente tratadas por ano foi calculado a partir da divisão do número de frascos de 300 mg adquiridos pelo número de frascos necessários ao tratamento de manutenção de um paciente adulto por ano (72 frascos). Foi utilizada ainda a dose diária definida (DDD), uma medida internacional de consumo recomendada pela OMS, que não tem relação estrita com a dose real e representa a dose média para um paciente adulto de 70 kg^15^ . A padronização pela DDD permite comparabilidade. Para estimativas populacionais, esse indicador é parametrizado como o número de DDD/1.000 habitantes/dia; neste caso, optou-se por apresentar o número de DDD/1.000 habitantes/ano^16^ . Os dados da população brasileira nos anos estudados foram obtidos do Instituto Brasileiro de Geografia e Estatística^17^ .

Todos os dados obtidos do Siasg foram compilados para um banco especificamente construído para esse fim, e os cálculos estatísticos, incluindo as análises de tendência, foram realizados utilizando-se o *software* Microsoft Excel^®^.

O acesso aos dados do Siasg é público, em conformidade com a Lei de Acesso à Informação, dispensando a submissão prévia do estudo a um comitê de ética em pesquisa.

## RESULTADOS

O mapeamento das compras do medicamento junto ao Siasg mostrou que as primeiras aquisições do eculizumabe ocorreram em fevereiro de 2009, dois anos após o primeiro registro internacional. No período examinado, todas as compras federais do eculizumabe foram realizadas pelo Ministério da Saúde, por meio do Departamento de Logística em Saúde, todas por dispensa de licitação e sempre com a justificativa de atendimento de demandas judiciais.

Houve uma tendência crescente no número de compras e nas quantidades adquiridas ao longo do tempo. Foram realizadas 283 compras no período, totalizando 116.792 frascos, 28,2% dos quais adquiridos em 2018 ( Tabela 1 ). Observa-se, ainda, que o ano de 2017 experimentou uma queda significativa no quantitativo de compras e de unidades farmacotécnicas compradas (13.721).

**Tabela 1:** Número de compras federais e de unidades farmacotécnicas adquiridas do medicamento eculizumabe (frasco-ampola de 300 mg), 2009–2018.

Ano	Número de compras	Unidades farmacotécnicas adquiridas	

		Quantidade	% Total
2009	2	145	0,12
2010	4	180	0,15
2011	12	754	0,65
2012	16	2.166	1,85
2013	69	8.296	7,10
2014	50	14.061	12,04
2015	57	19.206	16,44
2016	54	25.386	21,74
2017	4	13.721	11,75
2018	15	32.877	28,15
**Total**	**283**	**116.792**	**100,00**

Nota: Não existiram compras federais nos anos de 2017 e 2018.

A Figura 1 mostra a relação entre o número de pacientes potencialmente tratados pelas unidades de eculizumabe adquiridas e o número de DDD/1.000 hab./ano, permitindo verificar proporcionalidade e tendência de progressivo aumento de ambos ao longo do tempo.

**Figura 1:** Estimativas do número de pessoas tratadas por ano e número de DDD por 1.000 hab./ano nas compras federais de eculizumabe, 2009–2018. DDD: dose diária definida

Os gastos contratados entre 2009 e 2018 totalizaram R$ 2,1 bilhões em valores correntes e R$ 2,44 bilhões em valores corrigidos pelo IPCA de dezembro de 2018 ( Tabela 2 ). Mesmo respondendo por mais de um quarto de todas unidades adquiridas no período, o ano de 2018 correspondeu a apenas 18,3% dos gastos do intervalo estudado ( Figura 2 ).

**Tabela 2:** Gastos contratados em valores correntes e corrigidos relativos às compras federais do medicamento eculizumabe (frasco-ampola de 300 mg), 2009–2018.

Ano	Gastos contratados valores correntes (R$)	Gastos contratados corrigidos^a^(R$)	% gastos totais corrigidos
2009	2.219.023,25	3.924.941,63	0,16
2010	2.366.823,72	4.000.091,69	0,16
2011	8.979.795,84	14.330.935,34	0,59
2012	28.783.166,97	43.131.746,38	1,76
2013	125.450.088,18	177.631.768,91	7,27
2014	213.729.757,88	285.742.220,30	11,69
2015	376.945.601,92	473.604.100,41	19,38
2016	624.621.376,84	709.126.486,07	29,02
2017	267.111.457,23	285.303.416,91	11,67
2018	447.156.839,74	447.156.839,74	18,30
**Total**	**2.097.363.931,57**	**2.443.952.547,39**	**100,00**

^a^Gastos corrigidos pelo índice nacional de preços ao consumidor amplo em valores de dezembro de 2018.

**Figura 2:** Proporções de unidades adquiridas e de gastos totais corrigidos com o medicamento eculizumabe nas compras federais durante o período de 2009–2018, segundo ano de compra.

Até o registro em 2017, observa-se grande variação no preço médio ponderado (PMP) pago por unidade farmacotécnica, que chegou a atingir o valor de R$ 27.933,76 em 2016. ( figura 3 ). As duas compras realizadas antes da obtenção do registro na Anvisa em 2017 (10.733 unidades, 78% do total comprado no ano) tiveram PMP de R$ 21.055,61 em valores correntes. Já as duas aquisições realizadas depois do registro e após a fixação do preço máximo de venda ao governo (totalizando 2.988 frascos) tiveram PMP inferior (R$ 13.515,01) ao estabelecido pela CMED em outubro de 2017 (R$ 13.614,90), mostrando impacto importante na redução (-35,8%). Todas as compras em 2018 tiveram PMP inferior (R$ 13.600,90) ao valor por frasco definido pela CMED em abril de 2018 (R$ 13.889,35).

**Figura 3:** Preço médio ponderado corrigido (em R$) praticado nas compras federais do medicamento eculizumabe (frasco-ampola de 300 mg), 2009–2018. Nota: Preços unitários corrigidos pelo índice nacional de preços ao consumidor amplo em valores de dezembro de 2018.

## DISCUSSÃO

O eculizumabe tem sido presença frequente nas páginas dos jornais e espaços das mídias nos últimos anos. O medicamento já foi descrito como o mais caro do mundo, com custo aproximado de US$ 410 mil por paciente/ano nos EUA em 2010^18^ . No Brasil, o custo de tratamento de um único paciente ao ano, obtido via judicial, ultrapassava R$ 800 mil em 2012^19^ .

Desde 2009, o eculizumabe vem sendo comprado no país em quantidades e gastos que mostraram tendência crescente ao longo do tempo. Em 2017, contudo, houve queda importante no número de compras (de mais de 50 compras nos três anos prévios para apenas quatro nesse ano) e de unidades adquiridas (uma redução de -49,5% em relação ao quantitativo adquirido em 2016). Os dados disponíveis no Siasg não permitem avançar no entendimento desse declínio abrupto, mas dois fatos podem ter contribuído. Em 2017, a Polícia Federal deflagrou a Operação Cálice de Hígia, com objetivo de investigar possíveis fraudes ligadas à judicialização de medicamentos para doenças raras, incluindo o Soliris^®^, nome comercial do eculizumabe^b^. Nesse mesmo ano, o MS empreendeu auditoria no processo de compra do medicamento. Das 414 pessoas que possuíam decisões judiciais para receber o fármaco em 2017, 28 não foram localizadas; cinco não residiam no endereço informado; seis se recusaram a prestar informações e 13 já tinham falecido. Mais grave: cerca da metade dos pacientes não apresentava provas de diagnóstico da doença e, ainda assim, vinha recebendo o eculizumabe por decisão judicial^c^.

Por outro lado, em 2018, houve retomada do crescimento em termos do número de compras e quantidades, com o ano sendo responsável por 28,2% do total de unidades adquiridas em todo o período estudado. Parte desse aumento pode ser reflexo da queda já mencionada em 2017, uma vez que se trata de medicamento de uso crônico, a ser utilizado por toda vida. Também não é incomum que a divulgação ampla do registro do medicamento produza um movimento de aumento da prescrição, no caso ainda sob a égide da judicialização, dado que o medicamento não havia sido incluído na tabela de pagamentos do SUS.

Essa inclusão só ocorreu no final de dezembro de 2018 e, até a data de elaboração deste manuscrito, o fármaco aguardava elaboração de protocolo de uso pelo MS para ser regularmente fornecido aos pacientes. Ao longo dos anos estudados e mesmo sem presença de registro ativo até março de 2017, o acesso ao medicamento ocorreu sempre por demanda ao Judiciário, em ações de caráter individual que têm sido responsáveis por gastos extremamente significativos.

A literatura aponta inúmeros exemplos de como o litígio tem sido utilizado como estratégia de acesso a medicamentos que não possuem registro no país^20^ . Evidências sugerem que empresas farmacêuticas podem usar de relações com grupos de defesa dos pacientes e profissionais de saúde para expandir a participação de mercado pela judicialização, terminando por forçar a incorporação do medicamento no sistema de saúde^23 , 24^ . Um estudo de 514 ações judiciais que demandaram o medicamento e tinham o MS como réu entre 2010 e 2016 mostrou que 376 (73%) tinham origem no Distrito Federal e 46 (9%) no estado de São Paulo. Apenas um único escritório de advocacia foi responsável por 361 processos (70%). As proporções de prescrições originárias de médicos particulares e do SUS são semelhantes (respectivamente, 32,4% e 31,2%), chamando atenção o fato de que, em 27,1% das ações, sequer existia registro do nome do prescritor^25^ .

O eculizumabe é considerado um “medicamento órfão”, o que lhe confere um conjunto de especificidades em termos de registro sanitário, preços e avaliações para pagamento ou reembolso pelos sistemas de saúde. A designação de “órfão” está associada a fármacos desenvolvidos para tratamento de doenças que presumivelmente proporcionariam pouco retorno econômico sobre o investimento em pesquisa e desenvolvimento (P&D) efetuado pela indústria farmacêutica ou detentores de patentes. A baixa rentabilidade pode se dever à baixa prevalência ou incidência da doença em uma população, o que tornaria o mercado para o medicamento pequeno e pouco lucrativo, ou ao foco terapêutico em condições prevalentes em países menos desenvolvidos e sem recursos para pagar o preço que representaria lucro para a indústria. Como resultado, inúmeras legislações foram sucessivamente criadas nos Estados Unidos (1983), Japão (1993), Austrália (1998) e Europa (2000) para incentivar o desenvolvimento dos medicamentos órfãos^26^ .

Chama atenção o elevado preço pago pelo eculizumabe nas compras do MS e, mais ainda, sua variação ao longo do tempo. O uso direcionado a uma doença rara é frequentemente relatado como motivo para o preço elevado do medicamento, com a justificativa de que incorre em custos de P&D semelhantes àqueles voltados a doenças comuns, mas com menor número de usuários potenciais para garantir o retorno dos investimentos. Essa justificativa, contudo, tem sido questionada. A venda de medicamentos para doenças raras tem se tornado por vezes mais lucrativa que a dos medicamentos tradicionais devido à concessão de diversos benefícios governamentais, como exclusividade de mercado por períodos mais longos, incentivos fiscais, apoio econômico para desenvolvimento de pesquisas específicas e aprovação para comercialização em tempo menor e com critérios de evidências científicas menos exigentes^26^ .

Entretanto, o custo dos ensaios clínicos para medicamentos órfãos é bastante inferior ao de drogas para outras doenças, porque o número de pacientes envolvidos é necessariamente muito menor. Pequenos estudos clínicos e ausência de tratamentos alternativos colocam esses medicamentos órfãos em vantagem, quando submetidos a revisões regulatórias. Poucos pacientes e falta de concorrência também determinam necessidade de menores investimentos em marketing. Além disso, pelo seu alto custo, esses medicamentos são geralmente financiados por governos ou seguradoras^29^ . Como resultado, empresas farmacêuticas com autorização de comercialização de produtos órfãos podem ser mais lucrativas que aquelas sem esse tipo de produto em seus portfólios. Entre 2000 e 2012, empresas que comercializavam medicamentos órfãos tiveram retorno do investimento 9,6% mais alto que os produtores de medicamentos não órfãos^30^ .

A solicitação de registro de medicamento no país e, após aprovação, o pedido de estabelecimento de preços pela CMED são atribuições exclusivas do fabricante. A ausência de registro e de preço determinou que, até 2017, o Ministério da Saúde era obrigado, para atender à demanda judicial de eculizumabe, a importá-lo a qualquer preço cobrado pelo fornecedor, arcando ainda com todos os custos de transporte e distribuição.

Ainda que o medicamento fosse objeto de compras por judicialização por longo tempo, a empresa não solicitou o registro no país por quase nove anos após sua obtenção em mercados como o americano e o europeu, mesmo considerando o tamanho da população brasileira e podendo contar com a priorização de análise dada aos medicamentos para doença rara pela Anvisa^31^ . Só o fez em 2015, ano em que coincidentemente terminava a proteção patentária do eculizumabe no país.

Sem o registro, não havia preço estabelecido para venda ao governo e nem se poderia submeter o medicamento à avaliação pela Conitec. Mas ele continuava a ser comprado via judicialização, sempre a preços elevados, com dispensa de licitação e para atendimento de pequeno número de pacientes. Ou seja, o ganho da empresa estava assegurado, com o produtor definindo e tendo, até então, os preços de venda sob seu total controle.

Esses aspectos tornam mais claro o efeito do registro sobre a redução de preços verificada, superior a 35% no ano de 2017, e a manutenção dessa queda, mesmo que em patamar menor, também em 2018. Vale destacar que o observado não foi consequência da incorporação pela Conitec, só ocorrida em dezembro de 2018 e a partir da qual se torna mais fácil a programação das necessidades e aquisições, permitindo compras regulares e centralizadas, economia de escala e a utilização do poder de compra do Estado para negociações e menores preços^32^ . Quedas maiores poderão ainda ocorrer, uma vez que a decisão de incorporação pelo SUS apresenta, como um de seus condicionantes explícitos, a “negociação para redução significante de preço”^7^ .

Outro fator com potencial de impacto redutor futuro é a possibilidade de estabelecimento de uma parceria de desenvolvimento produtivo (PDP), para transferência tecnológica e produção local, uma vez que o medicamento não está mais sob patente. A esse respeito, destaca-se que o eculizumabe fazia parte da lista anual de produtos estratégicos para o SUS com vistas à formação de PDP publicada na Portaria MS nº 704, de março de 2017^33^ . O medicamento não consta, contudo, da Portaria MS/GM nº 731, de 26 de março de 2018,^34^ que traz a lista de projetos aprovados, ou da lista das propostas de PDP não aprovadas disponibilizada na página do DECEIIS/SCTIE^d^, levando a supor que não foram apresentadas propostas por empresas interessadas.

Por outro lado, embora seja esperada queda nas demandas judiciais após a incorporação ao SUS, ela não necessariamente ocorrerá. A incorporação estabelece um mercado oficial, no entanto, com preços mais baixos que os praticados na judicialização e apenas para a indicação definida (HPN). Ainda é possível o recurso à via judicial para os casos que não atendam aos critérios presentes no protocolo de uso, bem como para outras indicações registradas (SHUa) e não registradas no país,^35 , 36^ mantendo vivo esse filão de ganhos para a empresa. Acompanhamento das compras e preços praticados poderá ser útil para melhor esclarecer o comportamento futuro relativo ao medicamento.

Os valores estimados de utilização, calculados de forma independente (inferidos pelo número de tratamentos potenciais ou calculados pela DDD/1.000 hab./ano) mostram-se coadunantes e com tendência progressiva de crescimento ao longo do tempo. Não há dados internacionais de uso em DDD, mas os calculados permitirão comparabilidade ao longo do tempo^37^ . As exigências do atendimento e tratamento dos pacientes apenas em hospitais que integram a Rede Nacional de Pesquisa Clínica e a obrigatoriedade do registro dos dados clínicos e farmacêuticos em sistema nacional informático do SUS poderão trazer dados mais confiáveis de utilização^7^ .

Algumas limitações do estudo merecem registro. Embora o Siasg apresente dados de compras contratadas e não de compras finalizadas, é razoável supor que, no caso de medicamentos adquiridos para atendimento judicial, essas compras tenham sido efetivadas. Os dados agregados presentes no Siasg também não permitem conhecer o número de pacientes tratados ou de novos atendidos a cada ano, as indicações de tratamento subjacentes às compras por judicialização e expansões de cobertura pós-incorporação. Por fim, o intervalo da pesquisa não permite identificar o impacto da incorporação ocorrida no fim do período estudado nas quantidades e preços praticados.

O crescimento dos volumes comprados indica expansão de uso do eculizumabe ao longo do tempo. Os altos gastos associados apresentam impacto financeiro significativo para a provisão de medicamentos pelo Ministério da Saúde, destacando-se que é um fármaco que até o final de 2018 tinha 100% do acesso vinculado à judicialização. Além disso, a variação dos preços praticados antes do registro também reforça a importância da exigência de registro aos fabricantes que desejarem comercializar medicamentos no mercado nacional. Finalmente, o estudo aponta para a necessidade de monitoramento e auditoria permanentes das compras de medicamentos para atendimento de demandas judiciais, mormente aqueles de alto custo.

## Footnotes

a
Ministério da Saúde (BR), Agência Nacional de Vigilância Sanitária, Câmara de Regulação do Mercado de Medicamentos-CMED. Preços máximos de medicamentos por princípio ativo, para compras públicas Preço Fábrica (PF) e Preço Máximo de Venda ao Governo (PMVG): tabela atualizada em 20/10/2017 [citado 27 mar 2019]. Disponível em: http://portal.anvisa.gov.br/documents/374947/2829072/LISTA_CONFORMIDADE_GOV_2017-10-20.pdf/d25373b5-3224-4ef5-b62f-9367de5fb8d0

b
Reuters. PF investiga fraude bilionária ligada a remédio para doença rara. Exame, 8 de maio de 2017. [citado 28 mar 2019]. Disponível em: https://exame.abril.com.br/brasil/pf-investiga-fraude-bilionaria-ligada-a-remedio-para-doenca-rara/.

c
Brasil. Ministério da Saúde. Ministério da Saúde adota medidas para garantir oferta de medicamentos para doenças raras. 07 de março 2018. [citado 01 abr 2019] Disponível em: http://portalms.saude.gov.br/noticias/agencia-saude/42719-ministerio-da-saude-adota-medidas-para-garantir-oferta-de-medicamentos-para-doencas-raras.

d
Ministério da Saúde (BR), Secretaria de Ciência, Tecnologia e Insumos Estratégicos, Departamento do Complexo Industrial e Inovação em Saúde. Propostas de Projeto de Parcerias para o Desenvolvimento Produtivo não aprovadas. Brasília, DF; 2017 [citado 4 abr 2019]. Disponível em: http://portalarquivos2.saude.gov.br/images/pdf/2018/junho/18/Propostas-de-projeto-de-PDP-nao-aprovadas-2017.pdf
